# Patterns and predictors of mortality in the first 24 hours of admission among children aged 1–59 months admitted at a Regional Referral Hospital in South Western Uganda

**DOI:** 10.1371/journal.pone.0312316

**Published:** 2025-01-02

**Authors:** Moses Ochora, Stella Kyoyagala, Lydia Kyasimire, Mercy Akambasisa, Margaret Twine, Muna Ahmed, Dan Lutasingwa, Dorah Nampijja, Elias Kumbakumba

**Affiliations:** Department of Paediatrics and Child Health, Mbarara University of Science and Technology, Mbarara, Uganda; University of Oulu: Oulun Yliopisto, FINLAND

## Abstract

Most deaths among children under 5 years occur within the first 24 hours of hospital admission from preventable causes such as diarrhea, pneumonia, malaria, and HIV/AIDS. The predictors of these deaths are not yet well documented in our setting. This study aimed to describe the patterns and predictors of these mortalities among children aged 1–59 months at a regional hospital in South Western Uganda. We conducted a prospective cohort study among 208 children aged 1–59 months admitted to Mbarara Regional Referral Hospital. The mortality rate within the first 24 hours was 7.7% (95% CI 4–12) and the median time to death was 7.3(2.62–8.75) hours. Most deaths occurred in infants, with severe pneumonia, severe acute malnutrition, and malaria as leading causes. Factors predicting mortality included admission during the night (AHR: 3.7, 95% CI 1.02–13.53, p-value 0.047) and abnormal neutrophil count(AHR: 3.5, 95% CI 1.10–11.31, p-value 0.034). The study highlights the importance of timely interventions, particularly for infants, and suggests extra monitoring for those admitted at night or with abnormal neutrophil counts.

## Introduction

The under-five mortality rate refers to the probability a newborn would die before reaching five years of age, expressed per 1,000 live births. About 4.9 million children under five years die annually, translating to 13,400 children dying daily before they make 5 years. This is happening despite the global progress in reducing under-5 mortality [1]. Globally, under-5 mortality has declined by 60% from 93/1000 live births in 1990 to 37/1000 live births in 2022. In Uganda, the under-5 mortality rate has dropped from 182.1 deaths per 1,000 live births in 1990 to 45.8 deaths per 1,000 live births in 2019 [1, 2]. Most of these deaths are from preventable causes like pneumonia, severe acute malnutrition, diarrheal diseases and malaria, in addition to newborn-related issues like prematurity and birth asphyxia [3, 4].

While numerous studies have documented the predictors of overall in-hospital mortality in children, there is limited research specifically focused on the first 24 hours of admission—a period that is crucial for survival. In particular, the clinical and demographic characteristics of children who die within this critical period are not well-documented in settings like ours in South Western Uganda. Although delays in emergency care have been implicated in these deaths, there is a significant gap in understanding all the contributing factors [5, 6]. Variables such as illness severity, comorbidities like severe malnutrition, and HIV status have been linked to in-hospital mortality but have not been rigorously studied as predictors of mortality within the first 24 hours of admission in our context [7, 8].This may not clearly bring out the fine degree of granularity required to study the patterns of children under five who die in-hospital.

Moreover, more than 50% of these deaths happen in the first 24 hours of hospital admission [5, 6]. These deaths are at times due to several factors including delays in seeking healthcare, inadequate healthcare interventions, financial limitations, lack of life-saving equipment, and insufficient support services, among others [9, 10]. Health workers in these settings often operate with minimal training and supervision, further complicating the delivery of critical care. This study, therefore, aims to describe the patterns and predictors of mortality within the first 24 hours of admission among children aged 1–59 months at a tertiary facility in South Western Uganda. Data generated from this study could guide the development of targeted interventions, improve early triage and management protocols, and ultimately reduce under-5 mortality.

## Methods

### Study design

We conducted a prospective cohort study among children aged 1–59 months admitted to the Pediatric ward of Mbarara Regional Referral Hospital (MRRH) from 13 July 2022 to 25 October 2022 and enrolled 208 participants.

### Study setting

The study was conducted in the Pediatrics ward of Mbarara Regional Referral Hospital (MRRH). MRRH is a regional referral situated in Mbarara City in southwestern Uganda, about 260 kilometers from Kampala, the capital city of Uganda. It is also a teaching hospital for the Mbarara University of Science and Technology (MUST) and other tertiary health training institutions in the region. MRRH receives patients from 13 districts in Southwestern Uganda and neighboring countries of Rwanda and the Democratic Republic of Congo. The Paediatrics ward of MRRH admits on average 5000 children annually.

### Inclusion criteria

Children who meet the following criteria were included in the study:

1. All acutely ill children aged 1–59 months admitted through the emergency room were enrolled in the study. Every admission was treated as a separate event and therefore even children being readmitted were enrolled in the study
2. Critically ill patients transferred from other wards like pediatric surgery and casualty for admission into the main pediatric ward were also included in the study.

### Exclusion criteria

1. Patients attending chronic follow-up clinics with stable disease who may be admitted were excluded. These children are occasionally admitted to wait for review in the follow-up outpatient clinics.
2. Cancer patients already in care admitted to the ward were excluded because they didn’t have acute illnesses but rather only came to receive chemotherapy
3. Patients admitted waiting for scheduled surgical operations or those admitted for particular investigations/work-up were also excluded from the study. These children do not usually require emergency treatment.
4. Stable patients transferred from other wards like Pediatric surgery or Accident and Emergency ward that are transferred for continuing hospital care were also excluded.

### Sample size estimation

To calculate the sample size for the predictors associated with mortality within the first 24 hours of admission, we used OpenEpi, an online epidemiological and statistical calculator. Using a similar study done in Muhimbili Hospital in Tanzania where the significant predictor of mortality was severe acute malnutrition as compared to normal nutrition status (24.4% Vs 10.8% Vs 9.0%, p = <0.001) [6], our required sample size was 208.

### Sampling procedure

We consecutively sampled all children who met the study criteria until the desired sample size was achieved.

### Study procedure

All critically ill children who had been triaged, admitted, and received emergency care were approached by a trained research assistant. The research assistant who was not part of the emergency admission team then screened the admitted children to check if they met the eligibility criteria, explained to the caretakers the purpose of the study, and obtained written informed consent from the caretakers who consented to participate in the study. The Principal Investigator, who is a practicing clinician in the Paediatrics ward then ensured that all the requisite study examinations and investigations were done. Consent and recruitment were done after the children had received the appropriate emergency treatment and care. The study team did not directly intervene in the care plans of the children and the investigations.

All admitted children aged 1–59 months who met the eligibility criteria were enrolled in the study after they were stabilized and followed up until the outcome which was either dead or alive at 24 hours post-admission. Pre-admission data regarding the time from onset of symptoms to contact with a health worker, previous diagnosis, home remedies, previous treatments, household status, and current referral status was taken. The time of arrival and time of review by health care workers, immunization status, presenting complaint, treatment instituted at home, working diagnosis, and investigations requested, treatment received, and duration of stay before the outcome was also recorded. The history of any previous illnesses and any emergency care given to the child was also recorded.

The clinical signs: respiratory rate, blood pressure, axillary temperature, level of consciousness, pulse-oximetry, and heart rate was taken and interpreted for age and sex. The anthropometry was done and converted to weight for age, weight for length/height and height for age assessed using the World Health Organization Child Growth Reference Standards [11]. A comprehensive systemic exam capturing emergency and danger signs like reduced level of consciousness, excessive vomiting or diarrhea, seizures, poor feeding, difficulty in breathing, etc. was recorded on admission as well.

Investigations done at admission included a Complete Blood Count, Random Blood Sugar, HIV Screening, and a Blood Slide for malaria parasites. All laboratory investigations were done at the MRRH main laboratory. The Principal Investigator double-checked all collected data for completeness and rectified any errors or omissions while the child was still in the Emergency Room. The study met all the costs of the investigations that were charged a fee or were not available in the hospital laboratory at any time during the study. All children admitted were managed by the clinical care team based on standard treatment protocols for the different conditions. The diagnoses were based on those made by the admitting team of which the principal investigator was not part of. For any death within 24 hours of admission, the study team provided psychosocial counseling to the caretaker and family members present at the time of death. Autopsies were not done to determine the cause of death and the study used the diagnoses at admission as proxies.

For the blood tests, 2 ml of blood was drawn under sterile conditions during the insertion of an intravenous cannula or a venipuncture. The area was swabbed with cotton dipped in 70% ethyl alcohol. The blood was collected in an EDTA tube for a complete blood count. A thin smear for malaria was made in the laboratory for microscopic analysis. Blood smears for malaria parasites were done using GIEMSA stain and examined by a laboratory technician using an Olympus CX21F21LED microscope.

For HIV tests, pre-test counseling was done, and a standard testing algorithm was used. Children under 18 months with positive rapid tests were referred for DNA PCR tests in the HIV clinic at MRRH. For the urine dipstick, the urine sample was analyzed within an hour of collection using Chemstrip-10A urine test strips. The random blood sugar was done on the bedside using one touch-type glucometer. The CBC was analyzed using Sysmex Automated Analyzer X5-10001. The lab results obtained were shared with the clinical team as soon as they were received.

### Study variables

#### Outcome variable

The outcome variable was death within the first 24 hours of admission.

#### Independent/Predictor variables

The independent variables were pre-hospital factors like maternal age, education level, income status, parity and birth order of the child, past medical history of the child, cultural and spiritual factors, and distance from home to hospital. The hospital factors were the child’s vital signs and anthropometry, laboratory findings, emergency care given, quality of reviews, diagnosis, and severity of illness.

### Data collection

We used a questionnaire to collect pre-hospital data from the caretakers of the children and a data abstraction tool for the demographic, anthropometric, clinical assessments, and laboratory data from the patient’s records.

All the children who were admitted were screened by a trained Research Assistant to check if they met the eligibility criteria. The Principal Investigator, who is a clinician in the Paediatrics ward then reassessed the patients and took off the samples for basic investigations and ensured that emergency care plans had been effected by the clinical care team. The Research Assistant enrolled those patients whose caretakers had consented to participate in the study. Consent and recruitment were only done after participants had received the appropriate emergency care and had been stabilized.

### Data management and analysis

We collected and managed the data using Research Electronic Data Capture (REDcap ™), a secure web-based data capture platform software used for research studies. Each Research Assistant used an android tablet with pre-installed REDcap to collect data. We checked all the collected data for completeness immediately after each recruitment. The data was cleaned and transferred to STATA version 14 for analysis.

For the first objective of describing the patterns of mortality (this refers to the time of death, age group of children under 5 dying, and conditions causing or contributing to their death), we used descriptive statistics like means, proportions, and modes and summarized the data in tables and graphs. The Gaussian assumption was assessed using the Shapiro-Wilk test and histograms. Where the data was not normally distributed, the median and interquartile range (IQR) were calculated. The Chi-squared test was used to compare the distribution of mortality within 24 hours of admission among the categorical variables.

For the second objective, bivariable analysis was done using cox-regression to identify crude associations between the exposure variables and the outcome. The measure of association between the exposure variables and the outcome variable i.e. mortality was hazard ratio together with its 95%confidence interval and the p-value. Then predictors with crude hazard ratios whose p-value is less than 0.2, and those that were biologically plausible were included in the multivariate Cox regression model to adjust for confounding effects. Predictors with adjusted hazard ratios having p-value <0.05 were considered statistically significant in the final considered multivariate model.

#### Quality control

A screening log was used based on the daily hospital admissions. A study management Standard Operating Procedures (SOP) tool was designed to monitor the entry and exit of patients into the study. All these tools were reviewed by the Principal Investigator, Research Supervisor, and Biostatistician. The tools were pre-tested before use to ensure that they were accurate and collected data that is relevant to answer the study objectives.

Anthropometric measurements, clinical examinations, and sample collection were done by the Principal Investigator and a trained Research Assistant. Weights and heights were done using the same weighing scale and height board respectively for all the patients. The weighing scales are routinely calibrated and standardized by the hospital maintenance department.

All children underwent the same procedure for laboratory investigations. All the tests were done at the Mbarara Regional Referral Hospital laboratory which is a certified laboratory. The hospital laboratory is certified to do these tests and they run daily internal quality controls and quarterly external proficiency quality control tests.

### Ethical consideration

Mbarara University of Science and Technology Research Ethics Committee (MUST REC) provided ethical clearance to conduct this study (IRB number: MUST-2022-451). Written informed consent to participate in the study was obtained from the caretakers of all the patients included in the study after receiving emergency care. We did not have any emergency room consent or deferred consent. Names and identifying information were not used and unique study codes were generated for each participant. Emergency care was given to all patients before recruitment. The laboratory tests carried out were shared with the clinical team and caretakers on the ward for timely patient care.

## Results

**Fig 1:**
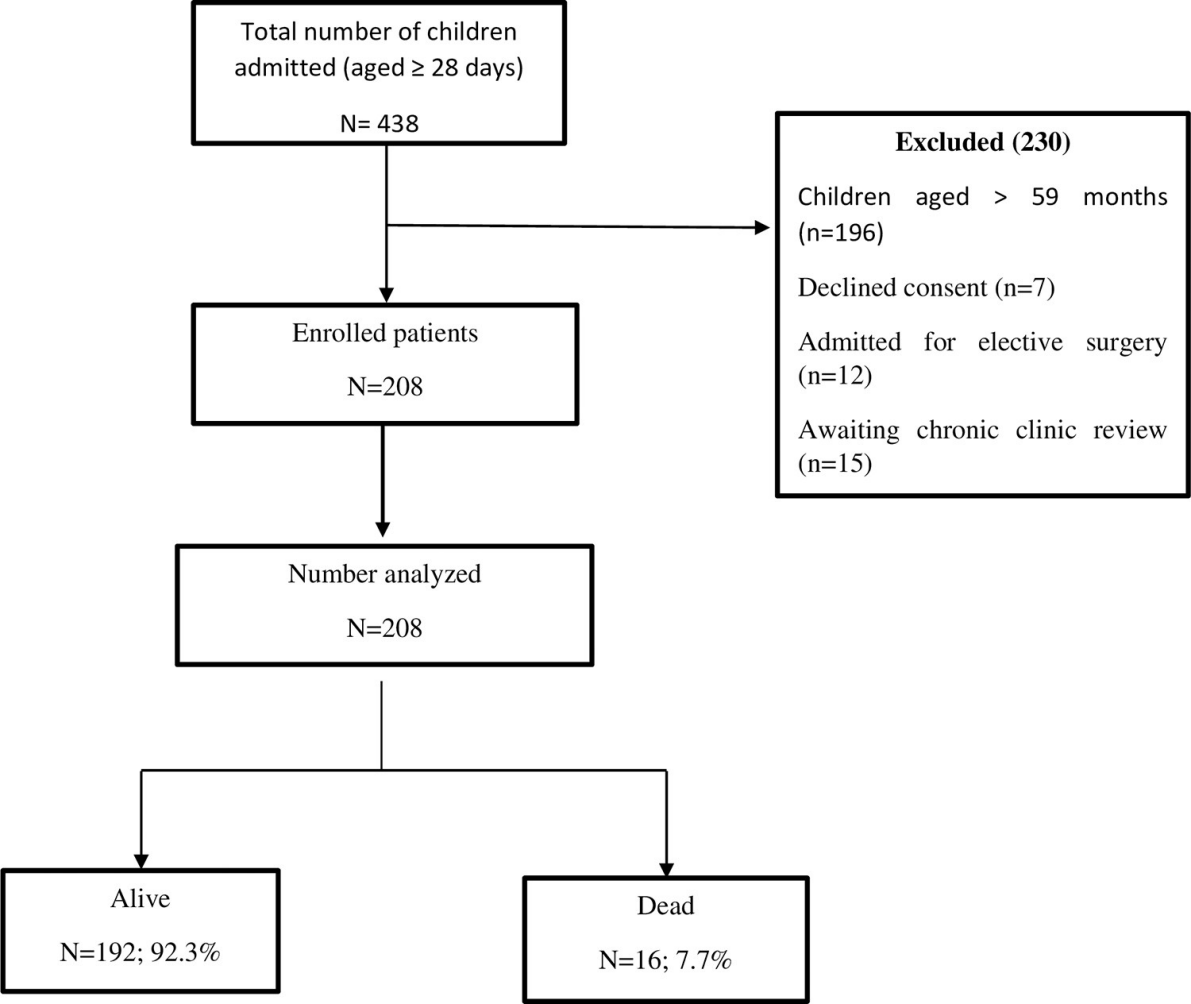


During the study period (July–August 2022), a total of 438 children above 28 days were admitted into the ward excluding cancer patients. Of these, 242 were aged 1–59 months. We excluded 34 children and enrolled and analyzed results for a total of 208 children aged 1–59 months. Five children of the 208 were readmissions and each readmission was treated as a different event (Fig 1).

### Baseline characteristics of children aged 1–59 months in the study

#### Baseline socio-demographic characteristics (Table 1)

The median age of participants was 13.0 (IQR 5.7–31.6) months. About one-third of the children had visited another facility before coming to MRRH. We admitted about one-third of the children during the weekend. Half of all children received emergency care within 30 minutes of arrival. More than one-third had symptoms for more than 48 hours before seeking health care.

**Table 1:** Baseline socio-demographic characteristics of the participants.

Variables		Total	Mortality		p-value
			Alive	Dead	
Sex	Female	93(44.7%)	85 (91.4)	8 (8.6%)	0.66
	Male	115 (55.3)	107 (93.0%)	8 (7.0%)	
Age (months)	1–11 months	95(45.7)	86(90.5)	9(9.5)	0.067
	12–59 months	113 (54.3)	106(93.8)	7(6.2)	
Relationship with caregiver	Mother	168 (80.8%)	155 (92.3%)	13 (7.7%)	0.96
	Other	40(19.2%)	37 (92.5%)	3 (7.5%)	
Primary caregiver’s Education	Primary	96(46.2%)	88 (91.7%)	8 (8.3%)	0.98
	Secondary	69(33.2%)	64 (92.8%)	5 (7.2%)	
	Tertiary	25(12.0%)	23 (92.0%)	2 (8.0%)	
	No formal education	18(8.6%)	17 (94.4%)	1 (5.6%)	
Tribe	Munyankole	141(67.8%)	131 (92.9%)	10 (7.1%)	0.64
	Others	67(32.2%)	61 (91.0%)	6 (9.0%)	
Day of admission	Working days	176(84.6)	163 (92.6%)	13 (7.4%)	0.70
	Weekend	32(15.4)	29 (90.6%)	3 (9.4%)	
Time of admission	Day	120(57.7%)	115 (95.8%)	5 (4.2%)	0.026
	Night	88(42.3%)	77 (87.5%)	11 (12.5%)	
Duration of symptoms	Less than 48 hours	93 (44.7%)	90 (96.8%)	3(3.2%)	0.030
	More than 48 hours	115 (55.3%)	102(88.7%)	13(11.3%)	
Health facility visit before MRRH	None	97(46.6%)	92(94.8%)	5(5.2%)	0.12
	Public facility	58(27.9%)	54(93.1%)	4(6.9%)	
	Private facility	53(25.5%)	46(86.8%)	7 (13.2%)	
Estimated waiting time	< 30 minutes ago,	118(56.7%)	108 (91.5%)	10 (8.5%)	0.70
	30–60	63(30.3%)	58 (92.1%)	5 (7.9%)	
	>60	27(13.0%)	26 (96.3%)	1 (3.7%)	
Remedies/medications	No	87(41.8%)	82 (94.3%)	5 (5.7%)	0.37
	Yes	121(58.2%)	110 (90.9%)	11 (9.1%)	

#### Clinical characteristics of children aged 1–59 months admitted at Mbarara Regional Referral Hospital (Table 2)

One-third of the children had a fever and altered level of consciousness. Half of the children had a normal nutritional status and less than a third were severely malnourished. One-third of the children had abnormal random blood sugar, low hemoglobin, and a high total white cell count.

**Table 2:** Clinical characteristics of children enrolled in the study.

Variables		Total	Mortality		p-value
			Alive	Dead	
Pulse rate	Normal	48 (23.1%)	45 (93.8%)	3 (6.2%)	0.55
	Abnormal	160(76.9%)	147(91.9%)	13(8.1%)	
Respiratory rate	Normal	66 (31.7%)	64 (97.0%)	2 (3.0%)	0.022
	Abnormal	142(68.3%)	128(90.1%)	14(9.9%)	
Temperature (°C)	Normal	123(59.1%)	118(95.9%)	5(4.1%)	0.08
	Low	85(40.9%)	74(87.1%)	11(12.9%)	
Weight-for-height	Normal	129(62.0%)	120(93.0%)	9(7.0%)	0.76
	Moderate	31 (14.9%)	29(93.5%)	2(6.5%)	
	Severe	48 (23.1%)	43(89.6%)	5(10.4%)	
Level of Consciousness	Normal	187 (89.9%)	175 (93.6%)	12 (6.4%)	0.039
	Altered	21 (10.1%)	17 (81.0%)	4 (19.0%)	
Clinical Pallor	Absent	172(82.7%)	162 (94.2%)	10 (5.8%)	0.026
	Present	36(17.3%)	30 (83.3%)	6 (16.7%)	
Jaundice	Absent	197(94.7%)	185(93.9%)	12 (6.1%)	0.007
	Present	11(5.3%)	7 (63.6%)	4 (36.4%)	
Oedema	Absent	184(88.5%)	170 (92.4%)	14 (7.6%)	0.90
	Present	24(11.5%)	22 (91.7%)	2 (8.3%)	
Dehydration	Absent	198 (95.2)	185 (93.4)	13(6.6%)	0.007
	Present	10 (4.8)	7(70%)	3 (30%)	
Hemoglobin level	Normal	138(66.3%)	127 (92.0%)	11 (8.0%)	0.83
	Low	70(33.7%)	65 (92.9%)	5 (7.1%)	
Neutrophils	Normal	115 (55.3%)	110 (95.7%)	5 (4.3%)	0.056
	Abnormal	93(44.7%)	82(88.2%)	11(11.8%)	
Blood smear for malaria	Negative	196(94.2%)	181 (92.3%)	15 (7.7%)	0.33
	Positive	12(5.8%)	11 (91.7%)	01 (8.3%)	
HIV test	Negative	199(95.7%)	185 (93.0%)	14 (7.0%)	0.094
	Positive	9(4.3%)	7 (77.8%)	2 (22.2%)	

#### Diagnostic categories of patients admitted (Table 3)

The most common diagnoses at admission were severe pneumonia (55, 26.44%), severe acute malnutrition (SAM) (49, 23.55%), sepsis (24, 11.54%), congenital heart disease (15, 7.21%) and malaria (12, 5.77%). For children with SAM, 9.1% had edema.

**Table 3:** Diagnoses at 24 hours of admission for children aged 1–59 months at MRRH.

MAIN DIAGNOSIS	NUMBER	Percentage (%)
Severe pneumonia	55	26.4
Severe Acute malnutrition	49	23.5
Sepsis	24	11.5
Congenital heart disease	15	7.2
Diarrheal diseases	13	6.3
Malaria	12	5.8
Acute CNS infections	8	3.9
Upper Respiratory Tract Infections	8	3.9
Hydrocephalus	6	2.9
Down syndrome	5	2.4
Poisoning	5	2.4
HIV related	4	2.0
Surgical conditions	4	2.0

### Patterns of mortality within the first 24 hours of admission among children aged 1–59 months admitted at Mbarara Regional Referral Hospital

#### Causes of death within the first 24 hours of admission among children aged 1–59 months admitted at Mbarara Regional Referral Hospital

**Fig 2:**
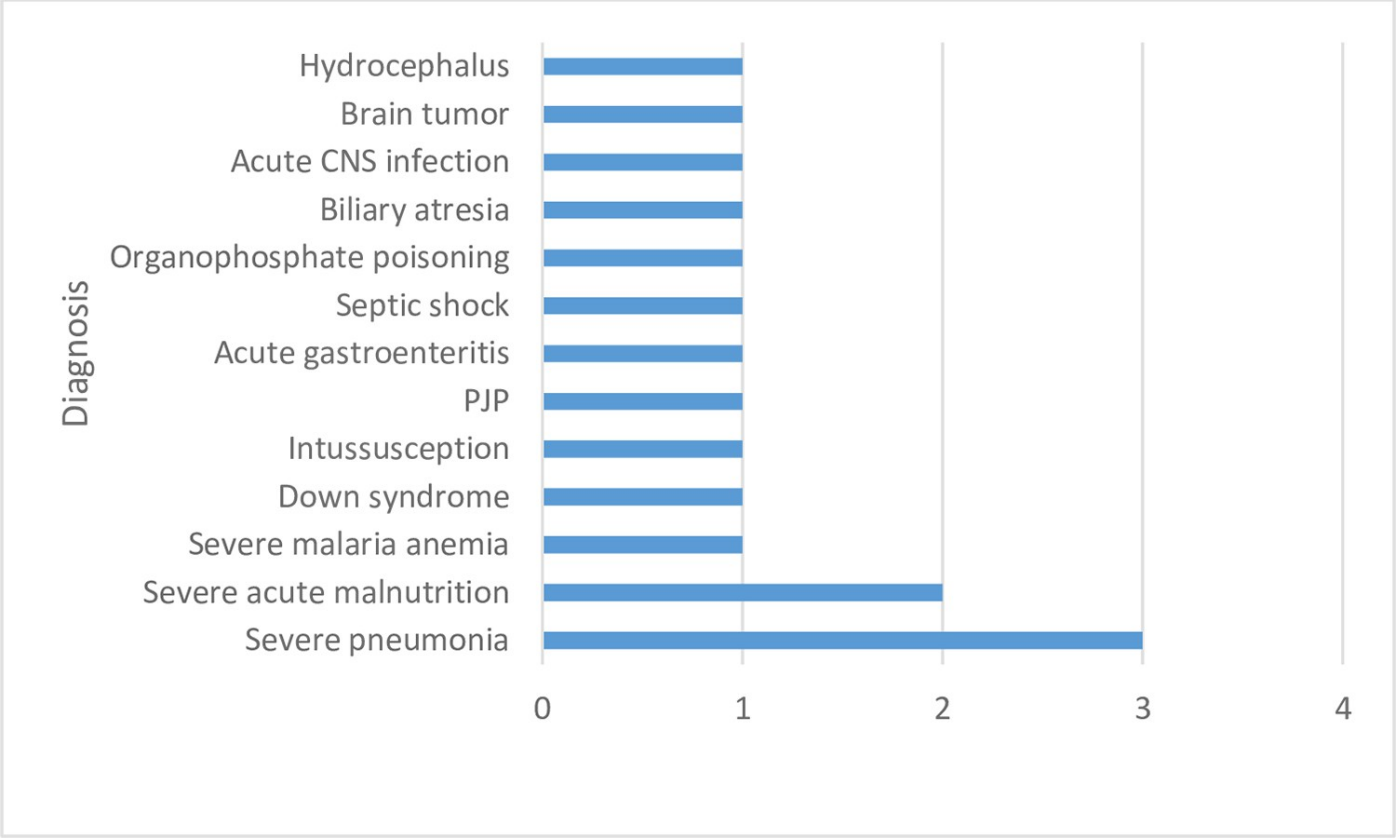


The common causes of mortality were severe pneumonia (3, 18.75%) and severe acute malnutrition (2, 12.5%) with case fatality rates of 5.5% and 4.1% respectively (Fig 2).

#### Other patterns of mortality within the first 24 hours of admission among children aged 1–59 months admitted at Mbarara Regional Referral Hospital

**Fig 3:**
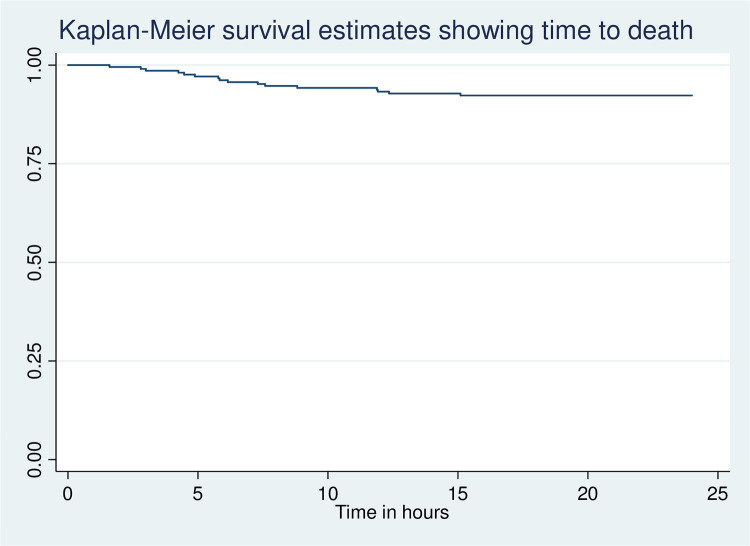


The overall mortality rate was 7.7% (95% CI 4–12) and the median time from admission to death was 7.3 (IQR 2.62–8.75) hours. More than two-thirds of children died within 12 hours of admission as shown in (Fig 3).

The mortality according to age categories was 9(56.3%) and 7(43.8%), children aged 1–11 months, and 12–59 months respectively. There was no sex difference among the children who died. The mortality was higher, 13 (81.3%) among children who sought care more than 48 hours after the onset of symptoms. Most children who died had visited another facility before coming to MRRH (11, 68.75%) and the majority had been to private facilities (7, 43.8%).

Most deaths occurred during the day shift (10, 62.5%) however the majority (11, 68.8%) had been admitted during the night shift. Ten (62.5%) of the children who died had received emergency care within less than 30 minutes of admission. Most of the children who died were admitted on a weekday (13, 81.3%).Severe pneumonia (3, 18.75%) and severe acute malnutrition (2, 12.5%) were the commonest causes of death.

### Predictors of mortality within the first 24 hours of admission for children aged 1–59 months admitted at Mbarara Regional Referral Hospital (Table 4)

**Table 4:** Multivariate analysis results of the predictors of mortality.

Variable	Category	CHR	p-value	AHR	p-value
Sex	Female	Ref			
	Male	0.8(0.31–2.18)	0.687	1.1(0.31–3.89)	0.887
Time of admission	Day	Ref			
	Night	3.1(1.08–8.97)	0.035*	3.7(1.02–13.53)	0.047**
Neutrophil count	Normal	Ref			
	Abnormal	2.8 (0.99–8.17)	0.053	3.5(1.10–11.31)	0.034**
HIV status	Negative	Ref			
	Positive	3.2(0.72–14.01)	0.126	2.3(0.40–13.584)	0.335
Respiratory rate	Normal	Ref			
	Abnormal	3.4(0.76–14.81)	0.108	2.1(0.425–10.2)	0.366
Temperature	Low	Ref			
	High	3.3(1.14–9.48)	0.027*	2.8(0.767–10.00)	0.12
Pallor	No	Ref			
	Yes	3.1(1.11–8.34)	0.031*	2.1(0.543–8.226)	0.28
Dehydration	No	Ref			
	Yes	2.7(1.01–7.28)	0.048 **	1.0(0.283–3.786)	0.958
Jaundice	No	Ref			
	Yes	5.5(1.56–19.66)	0.008**	1.6(0.23–10.986)	0.634

CHR-Crude Hazard Ratio, AHR- Adjusted Hazard Ratio

*** p < .01

** p < .05

* p < .1

At multivariate analysis using stepwise regression and adjusting for different confounders, the hazard of death was directly proportional to being admitted during the night shift (AHR: 3.7, 95% CI 1.02–13.53, p-value 0.047), and abnormal neutrophil count (AHR: 3.5, 95% CI 1.10–11.31, p-value 0.034).

## Discussion

### Patterns of mortality within the first 24 hours of admission

The mortality rate within 24 hours of hospital admission among children aged 1–59 months in this study was 7.7%. This is still higher than the SDGs and WHO target of below 25/1000 [12]. This high rate means that the emergency care offered to children who present to our units needs to be improved. Many such children may present in very critical conditions, have multiple comorbidities, and may be difficult to resuscitate. Studies in Uganda [6], and Nigeria [13] have also found rates between 5–10%. However, These rates are still higher than global estimates of 4.3%. Many high-income countries have rates of 0.5–1.5%, indicating more robust and better-developed emergency care. Many LMICs still have challenges in their healthcare systems like late referrals, reduced health worker coverage at night, and inadequate emergency supplies [3]. It is, however, much lower than in other African countries where the 24-hour mortality rate ranged from 30% to 67% [14, 15]. The studies that were done by Lahmini et al. and Jofiro et al. in Morocco and Ethiopia, respectively, included neonates and neonatal mortality disproportionately increased the mortality rate. The higher rates observed across these studies could also be due to differences in the study design, where these studies used a retrospective study design and could have missed a lot of vital data [14, 15]. The Department of Paediatrics at MRRH needs to improve its emergency care steadily by increasing the number and expertise of emergency admission teams and creating a better-equipped emergency room.

The median time to death in our study was 7 hours, and only one child died within an hour of admission. This highlights a gap in emergency care given to children under 5 in low-resource settings since 7 hours should provide ample emergency care and institute long-term care plans. There is a lack of literature on time to death within 24 hours of admission for children in the age group of 1–59 months. However, A study done in Morocco had a median time to death of 12 hours and 10% of the children died within the first hour of admission [15]. This study recruited newborns who have higher odds of dying commonly due to prematurity, hypothermia, infections, and birth asphyxia.

This study also showed more deaths among infants as compared to toddlers. Infants have an immature immune system compared to toddlers making them more susceptible to severe infections. It is also more difficult for caregivers to identify danger signs among infants as they may not express themselves as compared to toddlers. This delays their access to health care and thus deterioration and increases the chances of death within 24 hours of admission. This finding means that keen attention should be given to infants as their illness manifestation may differ from older children and the severity may not be easily classified by a naïve person. This is similar to other studies done in Uganda [6], Morocco [15], and Ethiopia [14] where infant mortality rates were between 2–12%.

More children with fever died as compared to those without fever. Fever is a marker of acute or chronic inflammation following pathogenic invasion. Fever patterns and grade indicate the infection’s severity and chronicity. In SSA, where infectious diseases like pneumonia, malaria, and diarrheal illnesses are very common, many children present with fever and are likely to deteriorate and die shortly after admission. This has been reported in several studies in sub-Saharan Africa, where high-grade temperatures are a major presenting complaint [10].

The most common causes of death in our study were pneumonia and severe acute malnutrition, and other infectious diseases like malaria and diarrheal diseases caused fewer deaths. These deaths were mainly complicated by HIV, sepsis, and shock, among other comorbidities. This is comparable to studies done in Uganda [6, 8]. These are all LMICs with a similar burden of infectious diseases. Malnutrition causes reduced cell-mediated immunity and humoral response making the children more susceptible to other infections like pneumonia. Many of these children also present late with septic shock and multiple organ dysfunction. However, this is different from studies done in high-income countries where noninfectious causes of hospital admissions were dominant. The difference in the burden of diseases seen between these HICs and LMICs like ours calls for comprehensive assessment, investigation, and management of comorbid disease conditions [16]. This also shows that a lot of children may present in very critical conditions with multiple comorbidities and may be difficult to resuscitate [16].

### Predictors of mortality within the first 24 hours of admission

Our study found an almost 4-fold increase in the likelihood of death within 24 hours of admission for children admitted at night compared to those admitted during daytime. More than half of the children died during the day but had been admitted during the night. The night duty cover is usually manned by few junior house officers who may not be as experienced and skilled enough to make appropriate critical decisions and provide emergency resuscitation and care. Late in the night, specialists and Senior House Officers are not physically present but can be consulted on the phone. This may not work in life-threatening emergencies that require meticulous evaluation and critical interventions to save a life. This results in deterioration and irreversible organ damage leading to death during the day. Many of the night admissions could also be due to late arrival from referral facilities or severely ill children. Similar observations have been made in studies done in Tanzania [5] and Brazil [17]. There is usually a smaller number of critically skilled staff working and also difficult to get emergency supplies of sundries at night [18]. However, a study done in India found no difference in mortality between day and night time admissions, and this was attributed to the strict treatment protocols in their Pediatric Intensive Care Units [19] Another systematic review and meta-analysis also found no difference in mortality between day and night time admissions [20]. On the contrary, a study done in Nigeria found more death among children admitted during the day which was explained by uncoordinated changes in nurses’ shifts that compromised care [21]. In our Paediatric department, there has been an improvement in the handover of patients during shift changes and also the utilization of a notice board where all priority patients are written.

We also found a 3.5-fold increase in the probability of death among children who had an abnormal neutrophil count. Neutrophils, as part of innate immunity, respond to pathogens and protect the body from infections alongside other immune system components. A high or reduced neutrophil count is associated with the depressed activity of other immune cells like the T-lymphocytes and natural killer cells in response to acute or chronic inflammation. This directly shows an overwhelmed immune system that cannot protect the body. Such responses are even worsened in infants and neonates with immature immune systems. In LMICs where bacterial infectious diseases are responsible for many hospital admissions, an abnormal neutrophil count strongly predicts early mortality. This has also been found in other studies conducted in sub-Saharan Africa [13, 22].

### Strength of the study

1. We used a prospective cohort study design, and we were thus able to infer causality to the identified risk factors associated with mortality within 24 hours of admission. We were able to collect the data required to reach our objectives.
2. We studied history, clinical examination, and laboratory factors and how they predict mortality within 24 hours of admission. Many studies have only looked at a few of these factors independently.

### Limitations

1. The range of investigations done at admission was limited to only CBC, Blood smear for malaria, random blood sugar, and HIV tests due to resource constraints. The inability to do other tests like blood cultures, chest x-ray, C-reactive protein, and DNA PCR of bacterial pathogens limited our ability to identify what other laboratory factors predict mortality in our setting.
2. Our study was conducted in a single setting. Despite being a regional referral hospital with a wide geographical catchment, our study results may not be relatable to those from a multi-center study or generalizable to other regions

### Conclusion

1. The mortality rate in the first 24 hours among children aged 1–59 months in MRRH is high at 7.7% (95% CI 4–12), the median time to death was 7.3 (IQR 2.62–8.75) hours, and death was higher among infants. Pneumonia and severe acute malnutrition caused the majority of deaths
2. Admission at night and an abnormal neutrophil count were predictive of mortality within 24 hours of hospital admission.

### Recommendations

1. We recommend that the Department of Paediatrics and Child Health reviews how protocols for the treatment of severe pneumonia and severe acute malnutrition are implemented by the health workers on the ward.
2. We recommend an audit of the night-time versus daytime practice to aid in the improvement of service provision with evidence
3. We recommend larger multicenter retrospective and prospective studies in the department/ Hospital and surrounding communities to investigate more pre-hospital/ community and laboratory predictors of mortality within 24 hours of admission.

## Supporting information

S1 File (DTA)
